# Optimising gold nanorods for photoacoustic imaging *in vitro*

**DOI:** 10.1039/c8na00389k

**Published:** 2019-02-12

**Authors:** Oscar B. Knights, Sunjie Ye, Nicola Ingram, Steven Freear, James R. McLaughlan

**Affiliations:** School of Electronic and Electrical Engineering, University of Leeds Leeds LS2 9JT UK J.R.McLaughlan@leeds.ac.uk; School of Physics and Astronomy, University of Leeds Leeds LS2 9JT UK; Leeds Institute of Medical Research, University of Leeds, St. James's University Hospital Leeds LS9 7TF UK

## Abstract

Gold nanorods (AuNRs) can be synthesised with different sizes but similar aspect ratios and therefore similar surface plasmon resonances (SPRs). Their strong optical absorbance governed by their SPRs facilitates their ability to be used as molecular-targeted contrast agents for photoacoustic (PA) imaging. The size of AuNRs has an effect on the PA conversion efficiency, melting threshold, and cytotoxicity, indicating that size can have a significant impact on overall biomedical efficacy. We investigated these factors for four different AuNRs (widths of 10, 25, 40 and 50 nm) all with SPRs of 815 ± 26 nm. A size-dependent linear relationship between fluence and PA amplitude was observed, along with particle melting. Reshaping was confirmed *via* transmission electron microscopy and spectrophotometry at a laser fluence of 11 ± 1.7 mJ cm^−2^, 20 ± 2.2 mJ cm^−2^, and 40 ± 2.6 mJ cm^−2^. Cytotoxicity was tested on lung cancer cells (A549) *via* a colourimetric assay at a maximum concentration of 3 × 10^10^ NP ml^−1^. Results demonstrate the 40 nm and 50 nm AuNRs produced the highest signal for equivalent particle numbers, but displayed the highest toxicity. Conversely, the 10 nm AuNRs were the most efficient photoacoustic converters, at equivalent total mass. This study demonstrates the importance of AuNR size and concentration on selection of AuNRs for their eventual clinical use.

## Abstract 2

A study to find the optimum sized gold nanorod for use in PA imaging and/or optical-based theranostics, by comparing four different sizes with similar aspect ratios.

## Graphical abstract

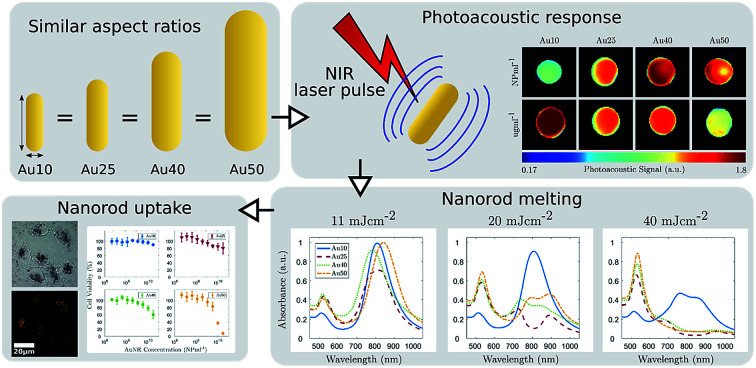

## 1 Introduction

Plasmonic nanoparticles^1,2^ have gained considerable interest over the past few decades due, in part, to their ability to efficiently absorb and scatter electromagnetic radiation.^3–5^ In particular, gold nanorods (AuNRs) possess characteristics such as tuneable surface plasmon resonances (SPRs),^6,7^ relative biocompatibility,^8,9^ high colloidal stability,^10–12^ comparatively easy synthesis,^13–15^ and functionalisation,^16,17^ that make them desirable for use in biomedical disciplines including diagnostics, therapeutics, or theranostics.^18–21^ Furthermore, their potential utilisation in nanomedicine as drug carriers, nano-probes, cellular labels, and biosensors is evident.^22–24^

With regards to diagnostics, AuNRs have shown to be effective contrast agents in photoacoustic (PA), or optoacoustic, imaging – a technique that relies on the photoacoustic effect.^25–27^ Since AuNRs can provide an enhanced optical absorption contrast against background tissue, a larger PA emission amplitude can be generated from highly localised and targeted areas.^28^ PA images are reconstructed by acquiring ‘A-lines’, *via* the scanning of a single-element transducer or the use of a phased array beamforming technique, and either used directly to form images – by determining time-of-flight and speed of sound measurements – or in more complex reconstruction algorithms such as back-projection.^29,30^ Multispectral optoacoustic tomography (MSOT) is another PA imaging modality that utilises the multiple spectral components of different tissues, where image contrast is achieved by exciting the tissues according to their unique absorption spectra.^31^ PA imaging is rapidly emerging as an effective alternative imaging modality for clinical use,^32,33^ but the use of AuNRs have limited clinical use currently.

The amplitude of the PA signals is important for two main reasons: firstly, PA waves are generally weak in magnitude (≤10 kPa) compared with other modalities that utilise ultrasound. For example, the typical focal peak pressures generated by clinical ultrasound scanners are in excess of 1 MPa. These low amplitude PA signals will consequently be reduced further (related to the amplitude attenuation coefficient *μ*_a_) as they travel through the various different tissues, caused by a combination of acoustic scattering, absorption, and mode conversions of the waves. This ultimately results in the rapid reduction in signal amplitude (*μ*_a_ = 0.3–0.5 dB cm^−1^ MHz^−1^ for an approximate soft tissue^34^), and the already weak PA signals may become undetectable. Secondly, a heightened PA amplitude, as a result of employing AuNRs, facilitates the use of significantly lower laser fluences to generate an equivalent PA response. In other words, lower-powered lasers could be used to achieve the same PA response while reducing the damaging effects that high energy laser exposures can cause. A safe exposure limit, known as the maximum permissible exposure (MPE) for skin,^35^ provides a potential upper-limit on the laser fluences that can be employed for diagnostic applications. Furthermore, it is difficult for light to penetrate deep into biological tissues, since significant optical attenuation is observed.^36–39^ Thus, there is a distinct problem with generating PA signals in biological tissue, especially with several centimetres of tissue between the light source and region of interest, and the optimisation of AuNR PA emissions could help to mitigate these problems.

Nevertheless, if these techniques were to be applied for the imaging of tumours located in areas such as the lung, then it may be possible to reduce the distance between light source and tumour by using a technique similar to endobronchial ultrasound.^40,41^ A laser fibre attached to an endoscope and passed through the mouth and into the lung could enable the irradiation of a tumour from within, leading to reduced optical attenuation. This also gives rise to the possibility for an ultrasound transducer to be located on the end of the endoscope, along with the laser fibre, and enable the detection of PA signals generated by the laser-irradiated AuNRs. Since cancer of the lung is one of the deadliest forms of cancer with very few diagnostic or treatment options,^42^ techniques such as PA imaging have the potential to be combined with other known therapeutic options, such as photothermal therapy, to create new ‘theranostic’ techniques for the simultaneous diagnosis and treatment of lung cancer.

The number of AuNRs used to generate a PA signal has a significant effect on PA amplitude, where an increase in AuNR number corresponds to an enhancement in PA amplitude.^43^ For future clinical use, optimising the concentration of AuNRs needed to produce a detectable signal is essential, since smaller doses could be administered to a patient and the toxicity to healthy tissues minimised. Functionalisation and targeting can help to increase the number of contrast agents that build up in a target region, however the resulting increase is often only moderate.^44^ It would therefore be beneficial if a sufficient PA amplitude was achievable without the need for a large number of AuNRs in the region of interest.

Plasmonic nanoparticles absorb light differently based on their size and shape,^45–47^ and it is well-known that the peak absorption wavelength of AuNRs, corresponding to the longitudinal SPR, is linearly proportional to their aspect ratio when the relative permittivity of the surrounding medium is constant.^48–50^ Consequently, if the shape is fixed and the aspect ratio is restricted to a small range of values to enable maximum absorption in the near-infrared,^51^ then this implies that there may be an optimal size for equivalent concentrations (NP ml^−1^) that maximises the PA emission amplitude. It is also important to consider the relative total mass (μg NP ml^−1^) of the AuNR solutions since a population of smaller sized AuNRs will have a smaller total mass at an equivalent number of particles to that of larger AuNRs. Confusion must not be made between the effects observed as a result of the change in volume over a change in concentration.

The size (or volume) of the AuNRs may also have an effect on a large range of other aspects, such as photothermal conversion efficiency,^52,53^ cellular interactions,^54,55^ and the biological immune response.^56^ Furthermore, the thermal stability of AuNRs is a major factor that can impact on the optical absorption efficacy, and therefore PA signal generation.^57–59^ If the AuNRs begin to melt under laser irradiation then their ability to absorb the incident laser-light will significantly diminish as the peak SPR band begins to blue-shift. There have been many reports on the reshaping, melting and fragmentation of AuNRs,^58,60^ but the quoted fluence reshaping threshold is often very different between sources. These differences are likely due to the large range of nanoparticle shapes and sizes studied. The biomedical application of AuNRs for PA imaging has been investigated extensively, however the literature is generally focussed on a specific nanoparticle composition,^61^ coating,^62,63^ shape,^64^ or application,^65^ and some of the underlying aspects of AuNRs have been overlooked. Thus, if AuNRs are to be translated into a clinical setting, it is crucial that there is a solid basis of understanding with regards to AuNRs and their complex biological, and optical interactions.

In this study, we tested four different AuNRs that have a similar aspect ratio but different sizes, in an effort to inform on their future use as contrast agents for PA imaging, or optical-based theranostics. PA emissions were recorded for each AuNR size at equivalent particle numbers and total mass, in addition to determining typical fluence thresholds for melting and their inherent cytotoxicity to a lung cancer cell line.

## 2 Material and methods

### 2.1 Photoacoustic signal amplitude from gold nanorods

**Fig. 1:**
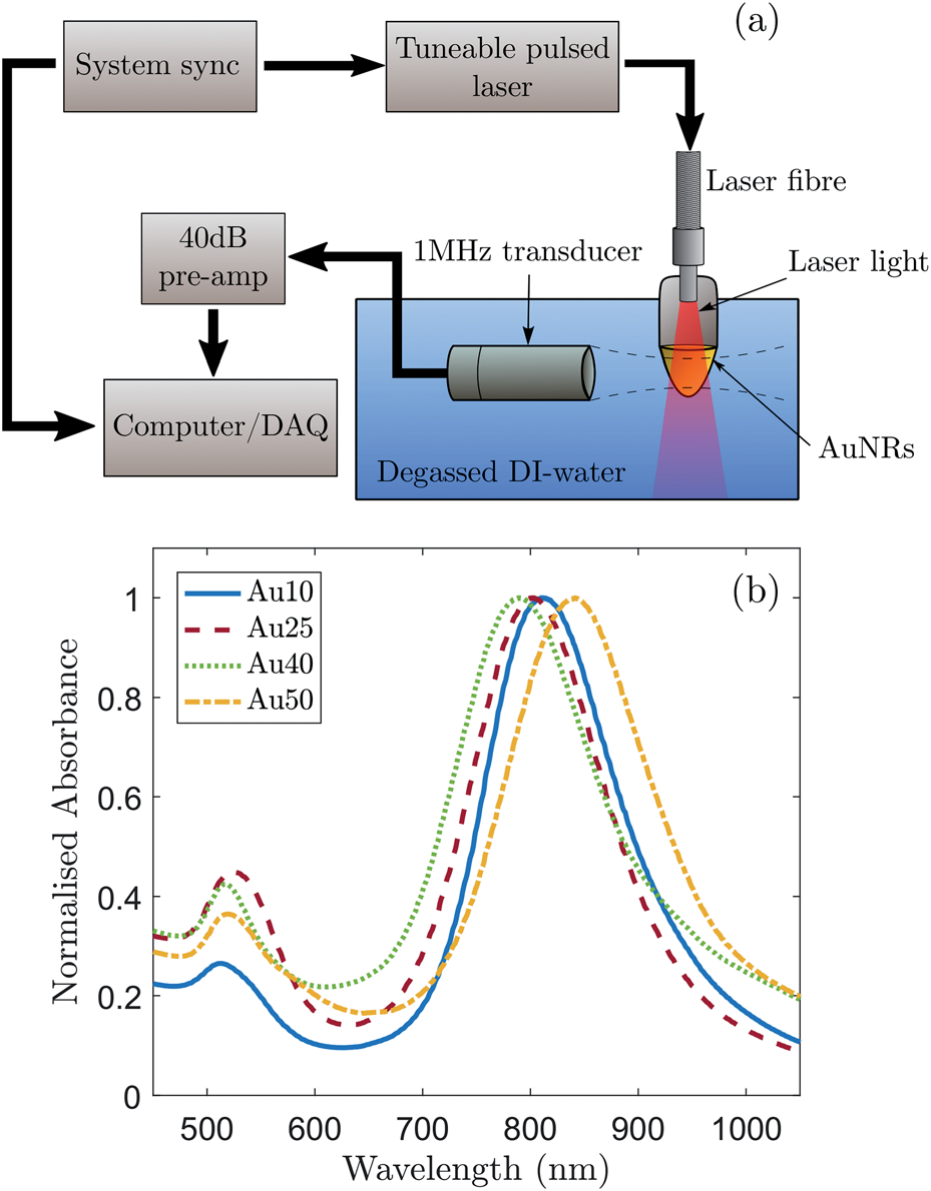
(a) A schematic of the experimental setup used to detect the photoacoustic signals generated from AuNRs with latitudinal widths of 10, 25, 40 and 50 nm along with the (b) normalised measured absorbance spectra displaying SPR peaks at 811 ± 2 nm, 803 ± 2 nm, 790 ± 2 nm, and 841 ± 2 nm, respectively. The SPR of the Au50s showed a 50 nm shift compared with that of the Au40s, which can be ascribed to a combined effect of the AuNR width and aspect ratio on the SPR position of AuNRs.

A schematic of the experimental setup for the detection of PA signals is shown in Fig. 1a. A pulsed tuneable laser system (Surelite™ OPO Plus, Continuum®, USA) operating at a pulse repetition frequency of 10 Hz with a pulse duration of 7 ns and spot size of 5 mm (at the focus of the transducer) was used to induce a PA response from a region. A single-element focussed transducer (V303, Olympus, UK) with a centre frequency of 1 MHz and certified −6 dB bandwidth of 76% was mounted on a micrometre translation stage and aligned to the centre of the AuNR solution. The detected acoustic signals were subsequently passed through a 40 dB pre-amplifier (SPA.1411, Spectrum, Germany) and recorded with a data acquisition (DAQ) card (M4i.4420x8, Spectrum, Germany).

Commercially bought, citrate-capped AuNRs (A12, Nanopartz, USA) with certified latitudinal widths of 10, 25, 40 and 50 nm were chosen as the source of the generated PA signals so that a relationship could be made between the PA response of AuNRs with different sizes, but similar aspect ratios. For clarity, the names Au10, Au25, Au40 and Au50 will be given to the AuNRs with certified widths of 10 nm, 25 nm, 40 nm and 50 nm respectively. Absorbance spectra of the AuNRs were measured and normalised (Fig. 1b) to determine the longitudinal SPR of each different sized AuNR. This enabled the laser wavelength to be tuned to the maximum SPR of each AuNR to ensure maximum optical absorption. A transmission electron microscope (Tecnai™ TF20, FEI, USA) was used to take images of the purchased AuNRs with an accelerating voltage of 200 kV and varying magnification. These images were subsequently analysed to confirm the size distributions and aspect ratios of the different AuNRs (Table 1). The manufacturer of the AuNRs characterise them before shipping and have stated that a minimum of 93% of the population of AuNRs across the four different sizes were rod-shaped. This was confirmed *via* TEM.

**Table 1:** Dimensions and SPRs of the AuNRs used in the study, determined by TEM analysis and absorbance measurements respectively. The uncertainty of measurement is the standard deviation of the sample

Name	Width	Length	Aspect ratio	Peak SPR
Au10	9.9 ± 1.1 nm	39.7 ± 5.4 nm	3.98 ± 0.51	811 ± 2 nm
Au25	23.2 ± 2.6 nm	85.6 ± 9.8 nm	3.73 ± 0.63	803 ± 2 nm
Au40	39.8 ± 4.1 nm	122.5 ± 13.8 nm	3.10 ± 0.35	790 ± 2 nm
Au50	42.2 ± 4.6 nm	142.0 ± 17.0 nm	3.38 ± 0.41	841 ± 2 nm

To ensure equivalent laser fluences were used to generate a PA response from each AuNR type, the output energy of the laser was measured across 100 pulses and calibrated for each wavelength used in the study. A solution of each AuNR type was made to a concentration of 1 × 10^11^ NP ml^−1^ by diluting a portion of the stock solution with DI-water. The AuNRs were agitated in an ultrasound bath for 15 min to ensure uniform distribution in the solution prior to being placed inside an Eppendorf for PA emission measurements. The incident laser fluence was increased sequentially from approximately 1–40 mJ cm^−2^ in steps equal to 4% of the highest laser output energy used.

To account for small fluctuations in the laser output energy and to increase the signal-to-noise ratio of the waveforms, the AuNRs received 20 pulses from the laser and the corresponding PA signals were averaged for each laser fluence. This was then repeated 3 times on fresh samples of each AuNR type. An increase in the total number of recorded averages only provided a minimal increase in signal-to-noise ratio of the signal and therefore the number of repeats was kept at 20 to reduce the total laser energy incident on the AuNRs.

To calculate the PA signal amplitude, a technique similar to that used in PA image reconstruction was used.^66–68^ Firstly, each averaged waveform was windowed to a 7 μs region of interest (ROI) that relates to the width of the absorbing volume. A Hilbert transform was then applied to obtain the envelope of each signal and the amplitude was calculated by integrating across the ROI. In addition to the AuNR PA signals, data was recorded and processed under the same conditions but with a water target instead of an AuNR solution so that the effects of the container could be accounted for by removing it from the calculated AuNR PA amplitude. The amplitude values determined per laser fluence from the three repeats were averaged and the standard error of the mean was calculated. Finally, a baseline noise signal was resolved by taking measurements under the exact same conditions except with the shutter closed while the laser was firing (*i.e.* no light was incident on the target).

The PA signal was also measured at the SPR of the four AuNRs using a pre-clinical multispectral optoacoustic tomographic (MSOT) system (MSOT inVision 128, iThera Medical, Germany). Four small straws containing equivalent concentrations of AuNRs (1 × 10^11^ NP ml^−1^) and another four with equivalent mass concentrations (100 μg ml^−1^) of the four AuNRs were made and inserted into a typical MSOT phantom (turbid agar phantom), along with a straw containing the supernatant of the AuNRs to act as a background signal. A multispectral scan (680 nm to 980 nm in steps of 5 nm) was performed at 6 unique points along the straws and the maximum PA amplitude at the peak SPR of the AuNRs was averaged to give a final amplitude. Images were processed using open-source software package ImageJ.^69^ In addition, a two-way analysis of variance (ANOVA) technique was used to calculate a *p*-value at each laser fluence studied to determine the statistical significance of the calculated PA amplitude of the AuNR with respect to the baseline signal amplitude.

### 2.2 Gold nanorod toxicity

To establish the cytotoxicity of the four different AuNR sizes, a 72 h MTT (3-[4,5-dimethylthiazol-2-yl]-2,5 diphenyl tetrazolium bromide) colorimetric assay protocol was followed.^70^ This metabolic assay provides an indication of cell viability by measuring the enzymatic activity of cellular mitochondria.^71^

A human non-small cell lung epithelial carcinoma cell line (A549, ATCC, UK) was cultured in DMEM (Dulbecco's Modified Eagle Medium) media supplemented with 10% FBS (Fetal Bovine Serum). When the cells reached 80% confluency, a 96-well plate was seeded with 1 × 10^3^ cells per well and incubated for 24 h. The four different sized AuNRs were introduced to the cells mixed with culture medium at a maximum concentration of 3 × 10^10^ NP ml^−1^ and each subsequent column of the 96-well plate received a 1 : 3 series dilution in concentration. One of the columns was reserved for a control group containing the supernatant of the AuNRs (DPBS) to ensure this was not the cause of toxicity. After 72 h incubation the AuNRs mixed with media was removed from each well and replaced with media containing MTT at a concentration of 500 μg ml^−1^. After a further 3 h incubation the media containing MTT was removed from each well and the 96-well plate was wrapped in foil and stored at approximately 4 °C, ready for absorbance measurements.

After the plates had been measured with a plate reader (Mithras LB 940, Berthold Technologies, Germany), the columns were averaged to obtain a single absorbance value for each AuNR concentration and the background absorbance level was then subtracted from each of the other values. The cell viability was finally calculated by the ratio of the mean absorbance of the sample with respect to the mean absorbance of the control group (DPBS). This methodology was repeated three times on fresh samples to obtain a mean cell viability.

### 2.3 Cellular uptake of gold nanorods

A549 cells were plated onto 22 × 22 mm glass cover-slips in a 6-well plate at a density of 1 × 10^5^ per well and allowed to grow for two days. The DMEM medium was then replaced with 2 ml of the same medium containing each AuNR type at a concentration of 1 × 10^11^ NP ml^−1^. After 4 h incubation, the AuNR-medium was removed and the cell monolayer on the cover-slip was twice-rinsed with DPBS (14190-094, Life Technologies, UK), fixed in 4% paraformaldehyde/DPBS for 10 min at room temperature and rinsed with DPBS twice. The fixed coverslips were mounted and sealed onto glass slides. Bright and dark-field microscopy imaging was performed with an inverted microscope (Nikon Eclipse Ti-E, Nikon UK Ltd, UK) and an oil coupled 100× objective (CFI Plan Fluor, Nikon UK Ltd, UK). Images were recorded with a 5 Megapixel colour camera (DS-Fi1, Nikon UK Ltd, UK) and saved using the NIS-Elements D software (Nikon UK Ltd, UK). Open-source software package ImageJ^69^ was used to crop and enhance the contrast of saved images.

## 3 Results and discussion

The determined size distributions (Table 1) for the Au10s, Au25s, and Au40s are in agreement with their corresponding peak SPRs,^50^ however there is a discrepancy with the Au50s. The Au50s were not measured to be 50 nm in width, as the certification suggested, resulting in a mismatch between the certified width and corresponding SPR (Fig. 1b). This discrepancy can be ascribed to the combined effect of the AuNR width and aspect ratio on the SPR position of AuNRs, and while the Au50s appear to be of a similar width to the Au40s, they are still larger in length and volume on average and so were still considered relevant for this study.

**Fig. 2:**
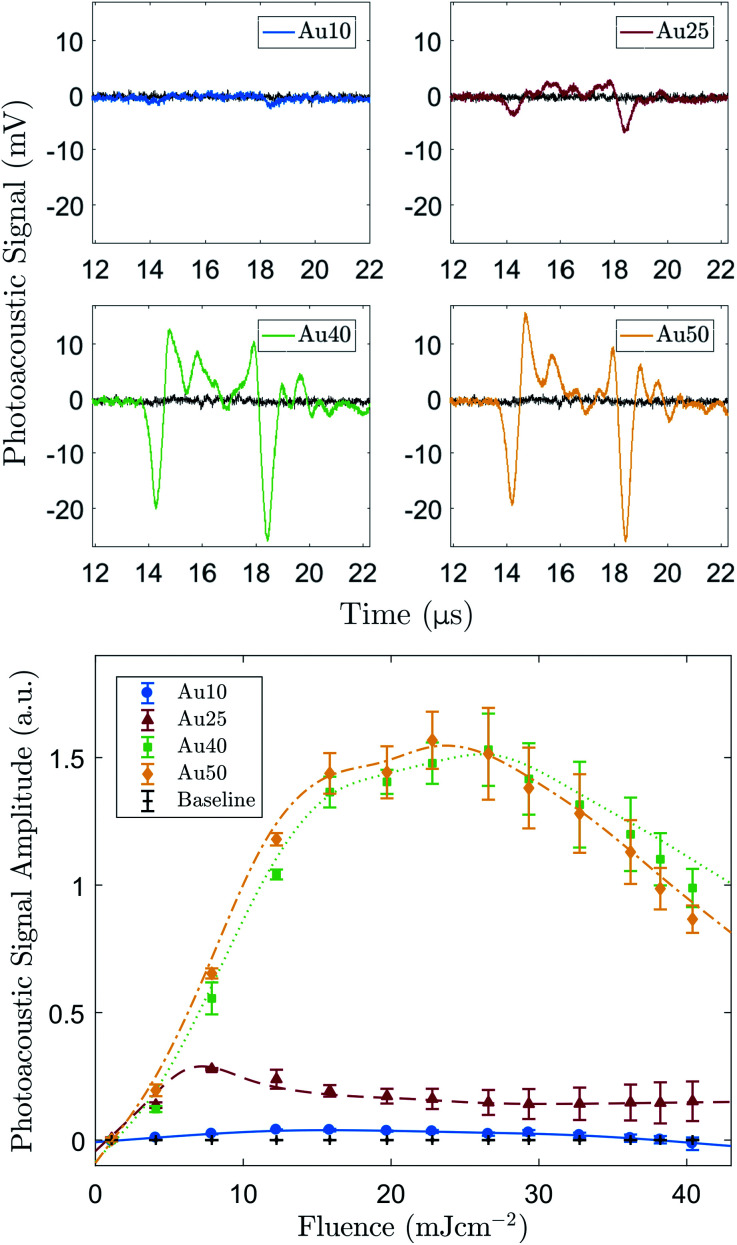
(a) Typical examples of the averaged photoacoustic signals (8 mJ cm^−2^ laser fluence) generated by all four AuNR sizes at a concentration of 1 × 10^11^ NP ml^−1^, detected with a 1 MHz focussed transducer, compared with the baseline signal (black). (b) Relationship between incident laser fluence and calculated photoacoustic signal amplitude generated from the same four AuNRs at a concentration of 1 × 10^11^ NP ml^−1^. Au10s (blue), Au25s (red), Au40s (green) and Au50s (yellow). The black line represents the water baseline signal.

Examples of the averaged recorded pre-processed photoacoustic signals are shown in Fig. 2a. The shape of the recorded photoacoustic signals can be explained by the high density of absorbers in the target region.^72^ The ROI was assumed to be cylindrical, with a radius of 1.3 mm (−6 dB radius of the transducer's focus) and height equal to 5 mm (spot size of the laser). The total ROI volume was approximately 26.5 μl, yielding an order of 1 × 10^10^ AuNRs converting the absorbed light into ultrasound. The large number of AuNRs collectively producing a photoacoustic response resulted in a pressure rise only at the outer edges of the absorbing region since the acoustic waves emitted from the centre interfered destructively.^72^ This explains the signal peak at 14 μs and 18 μs.

Fig. 2b shows the PA amplitude of each AuNR type as a function of the incident laser fluence. The PA signals from all four AuNRs were detectable above the noise at the concentration studied, however the Au10s produced a very weak signal in comparison. Statistical significance was established for the majority of fluence levels (*p* < 0.05), however there were a few instances where this was not the case. Firstly, at the lowest laser fluence studied, 1 ± 0.7 mJ cm^−2^, the amplitude of emissions from all AuNR sizes were not significantly greater (*p* > 0.05) than the baseline measurements. This can be attributed to the laser fluence being too low to generate a sufficient PA signal from the AuNRs. For the Au25s measurements (Fig. 2b), when the fluence reached and exceeded 29 ± 3.2 mJ cm^−2^, the detected emissions were not significantly above the baseline. Similarly, for the Au10s, this occurred at a laser fluence of 33 ± 3.3 mJ cm^−2^. The reduction in the significance of the data can be ascribed to the reduction in PA amplitude due to the potential melting or reshaping of the AuNRs.

All AuNR types displayed a linear relationship between incident laser fluence and PA amplitude at relatively low energies (<12 mJ cm^−2^ for the Au10s, Au40s, and Au50s, and <8 mJ cm^−2^ for the Au25s). The Au25s were the first to show a decline in signal amplitude, occurring when the fluence rose above 7 ± 1.9 mJ cm^−2^. Conversely, the PA amplitude of the Au10s, Au40s, and Au50s continued to increase past 7 mJ cm^−2^ and lose linearity at approximately equivalent fluences (12–16 ± 2 mJ cm^−2^). An explanation for this may be that the thermal stability of AuNRs is governed by a balance between the rate of heat dissipation to the surroundings and the atomic surface diffusion of AuNRs. It has been suggested that thermal stability significantly decreases with increasing aspect ratio,^60^ and this agrees with the observed results where the Au25s (aspect ratio = 3.73 ± 0.63) displayed a lower thermal stability to the Au40s (aspect ratio = 3.10 ± 0.35). The Au10s may show an enhanced thermal stability despite having a larger aspect ratio (3.98 ± 0.51) as they are much smaller than the other AuNRs and are able to dissipate the generated heat more rapidly to the surrounding environment.^59^ It is worth noting that the quoted fluence levels here are all below 31 mJ cm^−2^ – the approximate maximum permissible exposure of skin for a single pulse (wavelength between 700–1400 nm).

The two larger AuNRs (Au40s and Au50s) both displayed a similar PA relationship with increasing laser fluence, but it was the Au50s that ultimately produced the largest peak signal. The similarity in PA amplitude between the two larger AuNRs is most likely due to the similarities in AuNR size distributions as confirmed by TEM analysis (see Table 1). Furthermore, these AuNRs continued to produce an increasing PA amplitude past the point where linearity is lost (12–16 ± 2.2 mJ cm^−2^) and began to decline in amplitude at approximately 25 ± 2.7 mJ cm^−2^. This was presumably due to there being a significant number of large AuNRs that had not fully reshaped and were still able to continue to absorb light.

**Fig. 3:**
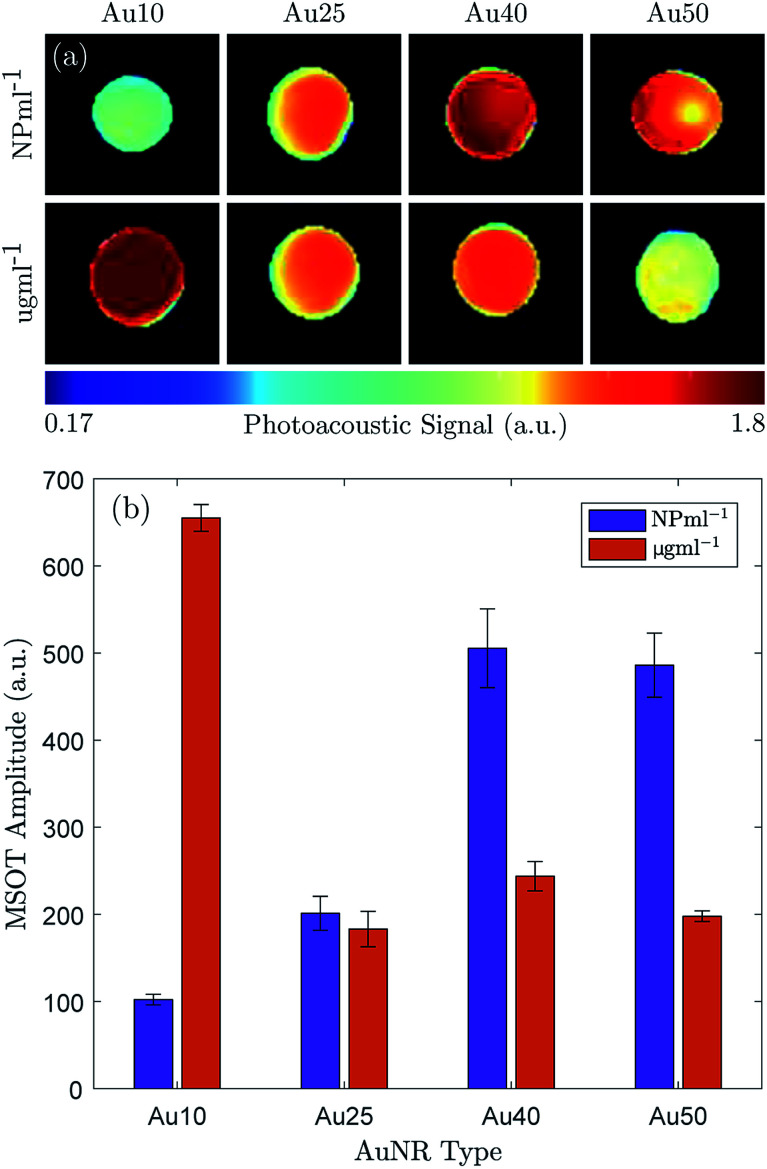
The photoacoustic response of the Au10s, Au25s, Au40s, and Au50s were measured with a multispectral optoacoustic tomographic (MSOT) system at equivalent mass (100 μg ml^−1^) and number of particles (1 × 10^11^ NP ml^−1^). (a) A MSOT image of the four AuNRs, reconstructed from the raw signal data captured with the MSOT system with a logarithmic colorbar, and (b) the maximum photoacoustic amplitude of the same four AuNRs (purple = 1 × 10^11^ NP ml^−1^, orange = 100 μg ml^−1^).

The maximum PA amplitude of the same four AuNRs was also measured using a MSOT system^31^ at a fixed number of particles (1 × 10^11^ NP ml^−1^) and fixed total mass (100 μg ml^−1^). The reconstructed PA images (linear regression) are shown in Fig. 3a and the PA amplitude data is shown in Fig. 3b. At equivalent particle numbers, the data is in agreement with the results in Fig. 2b, where an increase in AuNR size produces an increase in PA emission amplitude, and the Au40s and Au50s showed similar PA amplitudes. However, when the total mass was fixed between samples the Au10s produced a significantly larger PA signal when compared with the other three. They exhibited an amplitude more than 2.5 times that of the Au40s, the next largest amplitude. The Au50s and Au25s produced a similar PA response, but the Au25s displayed the lowest photoacoustic conversion overall.

At an equivalent total mass of 100 μg ml^−1^ the number of particles within each sample varied significantly. The Au10s contained more than an order of magnitude more particles (1.57 × 10^12^ NP ml^−1^), the Au25s were approximately the same concentration (1.57 × 10^12^ NP ml^−1^), the Au40s contained 3 times fewer particles (3.31 × 10^10^ NP ml^−1^) and there were more than 5 times fewer Au50s (1.82 × 10^10^ NP ml^−1^). This agrees well with the observed data.

The results indicate the importance of not only the AuNR size for PA emissions but also on how the concentration of AuNRs is defined. There is a clear distinction between the effects generated by a total number of particles and a total mass of gold, and this must be taken into consideration for tumour targeting, accumulation, and uptake of the AuNRs in a clinical setting. For instance, if the accumulation of AuNRs *in situ* is restricted by the total number of AuNRs, regardless of size, then the data would suggest that the larger AuNRs would be more effective at achieving the desired effects since they produce the strongest PA signal per particle (see Fig. 3).^73^ Conversely, if the total mass is the driving factor behind tumour uptake, then clearly the smallest AuNRs (Au10) would be most suited.^74^ However, it appears that the Au25s should be avoided in either situation since they produced the weakest PA signal in all cases.

**Fig. 4:**
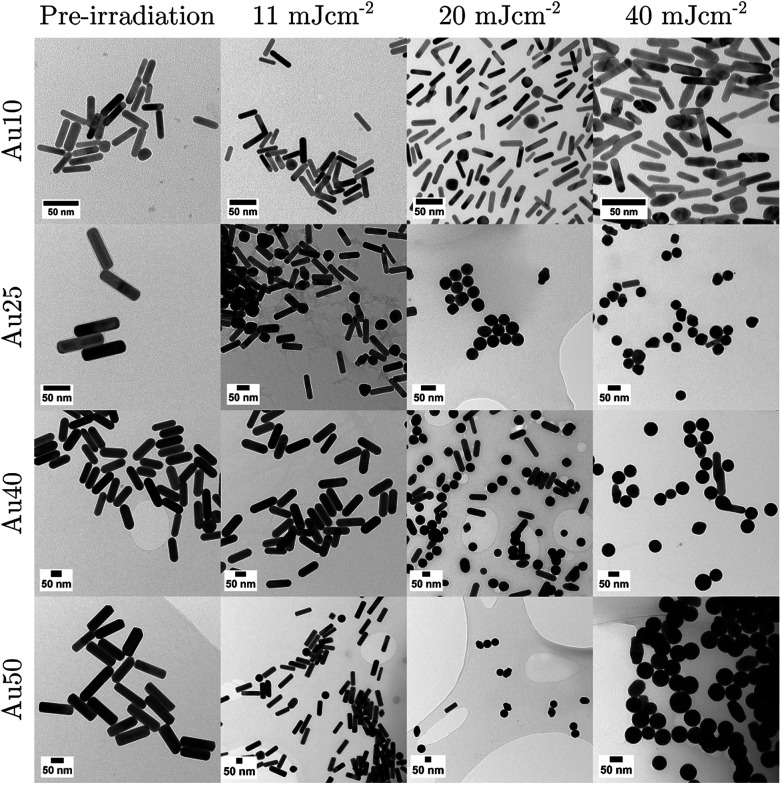
TEM images taken for each sized AuNR at specific points in the study: before laser irradiation, and after 20 pulses at 11 mJ cm^−2^, 20 mJ cm^−2^, and 40 mJ cm^−2^. Scale bars = 50 nm.

**Fig. 5:**
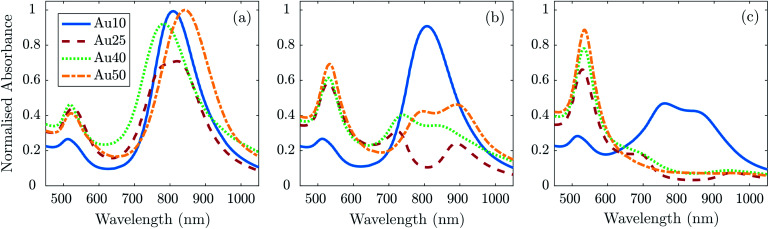
Absorbance measurements normalised to the maximum of the absorbance spectra taken before laser irradiation (Fig. 1a) for each AuNR size at a concentration of 1 × 10^11^ NP ml^−1^ after exposure to 20 laser pulses at a fluence of (a) 11 ± 1.7 mJ cm^−2^, (b) 20 ± 2.2 mJ cm^−2^, and (c) 40 ± 2.6 mJ cm^−2^.

To confirm if the reduction in amplitude with increasing fluence (and subsequent increase in calculated error) was a result of the AuNRs beginning to melt and reshape, thus causing a reduction in optical absorption (at each particles SPR) and photoacoustic emission; TEM images (Fig. 4) and absorbance measurements (Fig. 5) were taken after the AuNRs were exposed to specific laser fluences at key points (11 ± 1.7 mJ cm^−2^, 20 ± 2.2 mJ cm^−2^, and 40 ± 2.6 mJ cm^−2^) across the range studied. Fresh samples of AuNRs were separately exposed to 20 laser pulses – the same number of pulses used in the PA study – to ensure the observed effects were not due to cumulative absorption. The absorbance spectra were normalised to the original spectra to highlight the change in SPR due to melting.

At 11 ± 1.7 mJ cm^−2^, the Au25s showed partial melting and reshaping while the other three showed no reduction or flattening of the peak absorbance (the minimal reduction of the Au40s is within error). After 20 pulses at 20 ± 2.2 mJ cm^−2^, there was a significant hole in the peak SPR of the Au25s, and the TEM images confirmed that the majority of the AuNRs had melted and reshaped into spheres. Both the Au40s and Au50s also displayed a flattening and minor hole in the peak absorbance at this laser fluence, whereas the Au10s maintained photostability with only a minimal reduction in peak absorbance. Finally, at a fluence of 40 ± 2.6 mJ cm^−2^, the Au25s, Au40s and Au50s all demonstrated melting and reshaping of almost the entire population of AuNRs. The Au10s exhibited a significant reduction in peak absorbance at this laser fluence, however a considerable number of Au10s appeared to have remained stable, indicated by a reduction of only half the peak absorbance. This would suggest that smaller AuNRs (widths < 25 nm) are more resistant to reshaping under laser illumination than larger AuNRs, and would further support the idea that small AuNRs are able to dissipate heat to the surroundings at a faster rate, compared with larger AuNRs.^75^

The photostability of AuNRs can be affected by a number of factors, including photothermal conversion efficacy, thermal conductivity, surface diffusion, AuNR defects, thermodynamic stability, and the coating surrounding the AuNRs.^76,77^ Furthermore, the optical absorption of the particles can have a significant effect on the reshaping of AuNRs. As the AuNR size decreases, the optical absorption of the particle will also decrease. This could suggest why the smaller AuNRs in this study were resistant to higher laser fluences. However, equivalent particle numbers of the different sized AuNRs results in the total mass within the absorbing region being different. Thus, a decrease in AuNR size will result in a decrease in total mass, which could result in a maximum (or minimum) of the mass-normalised optical absorption for different sized AuNRs, and suggest why the Au25s appear to be significantly less photostable than any of the other AuNRs studied.^46^ The melting-point depression phenomenon may also contribute to the reshaping thresholds of small AuNRs, nevertheless the overall thermal stability is mostly governed by a balance between the total absorbed light and the thermodynamic stability of the AuNRs.^78,79^

In addition to the large reduction in absorbance at the longitudinal SPR of the AuNRs, a noticeable increase in the absorbance at the latitudinal SPR is observed (see Fig. 5). This can be explained by an ever-increasing number of AuNRs reshaping into spheres (see Fig. 4), as confirmed by TEM. The Au10s were the only exception, where the absorbance around 532 nm does not appear to increase despite the reduction in the 811 nm peak. TEM analysis confirmed that the majority of Au10s had not fully reshaped into spheres but had instead become *ϕ*-shaped or imperfect spheres. Fig. 5c also appears to support this idea as the spectra around the peak SPR broadens, indicating a distribution of particle shapes between a rod and sphere. This may be due to small AuNRs exhibiting a higher thermal coupling to the surrounding environment and therefore enabling a rapid dissipation of heat to the surrounding area, solidifying before becoming completely spherical.

Knowledge of the way in which AuNRs melt and reshape under laser illumination is crucial to the development of the modalities that rely on them. Slight changes in their size and shape can have a substantial effect on the optical interactions exhibited by AuNRs. If melting occurred during PA tomography or any other optical-based diagnostic or therapeutic modality that relies on AuNRs, the quality and efficacy of the technique would diminish significantly as a consequence. Thus, when selecting AuNRs for clinical use, the effect that AuNR size has on optically-induced degradation is an important aspect to consider.

**Fig. 6:**
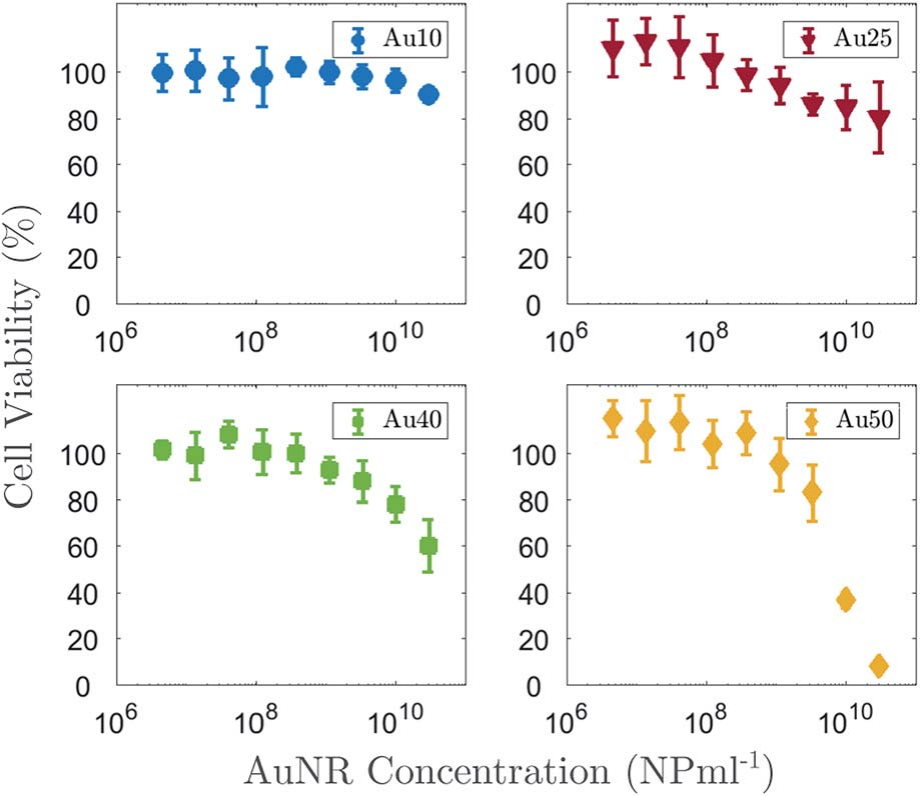
Percentage viability of an A549 lung cancer cell line after 72 h incubation with Au10s (blue, top-left), Au25s (red, top-right), Au40s (green, bottom-left) and Au50s (yellow, bottom-right) at a maximum concentration of 3 × 10^10^ NP ml^−1^.

In addition to understanding how the size of the AuNRs affects the induced PA response, it is also important to determine the impact that size has on cellular toxicity. Fig. 6 shows the percentage viability of a non-small cell lung cancer cell line (A549) after 72 h exposure to all four AuNRs across a range of concentrations from 1.5 × 10^6^ NP ml^−1^ to 3 × 10^10^ NP ml^−1^. As expected, the data suggested that an increase in the concentration of AuNRs also resulted in an increase in cytotoxicity. The concentration at which cell viability drops to 50%, known as the IC_50_, could not be deduced for the Au10s or Au25s since the concentration used in this study did not reach a high enough level to cause major detriment to the cells. A four parameter logistic regression curve was fitted to the Au40 and Au50 data to enable the IC_50_ to be determined.^80^ The Au40s displayed noticeable toxicity to the A549 cells, however an IC_50_ value (IC_50_ = 6.6 × 10^10^ NP ml^−1^) higher than the maximum concentration studied, was extrapolated from the regression curve. The Au50s demonstrated the highest toxicity of the four AuNR types, where a concentration of 3 × 10^10^ NP ml^−1^ resulted in necrosis or apoptosis of almost the entire cell population. An IC_50_ of 7.3 × 10^9^ NP ml^−1^ was easily deduced for this AuNR type. The data would suggest that larger AuNRs exhibited a higher toxicity at equivalent concentrations compared with that of smaller AuNRs. It is important to note that at equivalent concentrations, a solution of large AuNRs contains more mass than a solution of small AuNRs.

**Fig. 7:**
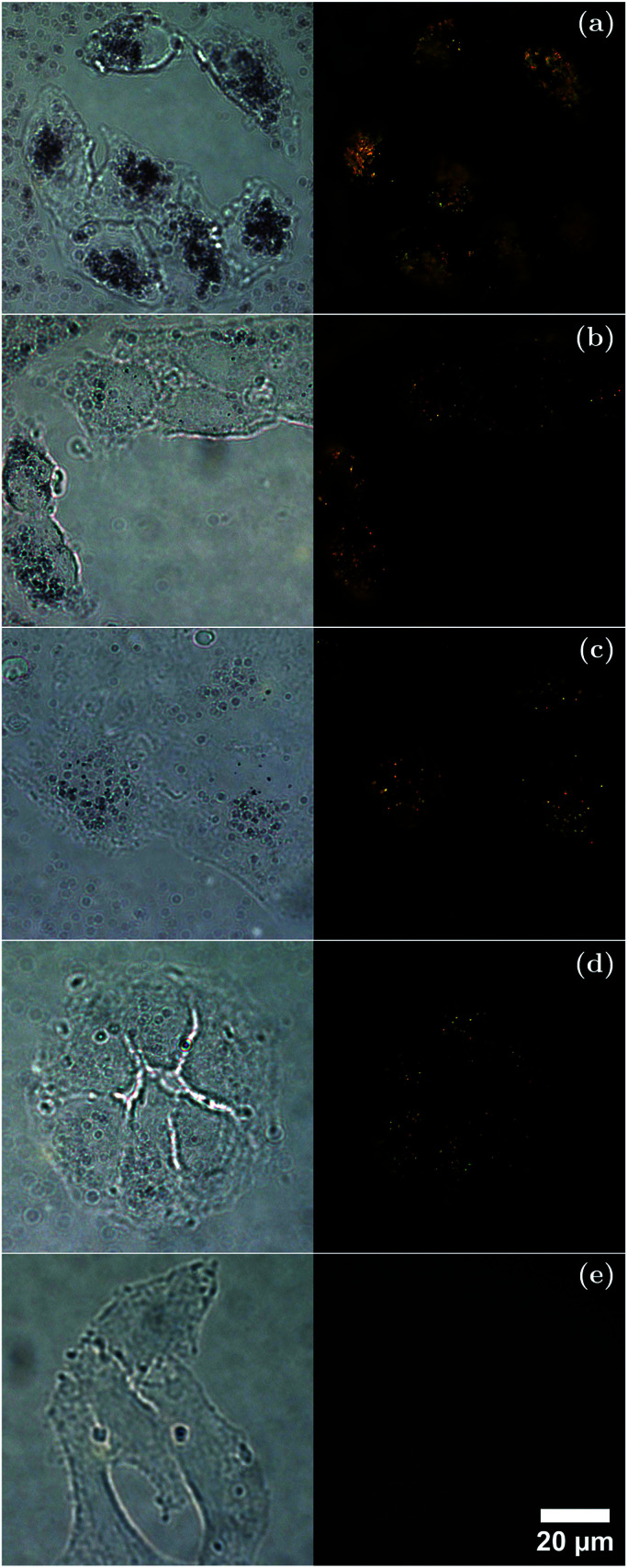
Bright-field (left) and dark-field (right) microscopy images of an A549 cell line after 4 h incubation in a medium containing (a) Au50s, (b) Au40s, (c) Au25s, (d) Au10s, and (e) control. AuNR images at a concentration of 1 × 10^11^ NP ml^−1^ for each AuNR type. All dark-field microscopy images are presented using the same brightness and contrast conditions. Scale bar = 20 μm.

To further understand the interactions between cells and the different sized AuNRs, bright-field and dark-field microscopy images were taken (Fig. 7) of A549 cells after incubation for 4 h in media containing each-sized AuNR. The bright-field microscopy images suggested that the cells incubated with AuNRs maintained their attachment to the glass slides and their normal morphology. It can be seen from the dark field microscopy images that the cells appear to have taken up AuNRs of each size, as observed by the scattered light. Careful examination of the dark-field microscopy images showed that the AuNRs enriched the cytoplasm of the cells instead of being evenly or randomly distributed on the cells, as would be the case for non-specific adhesion.^81^ While dark-field microscopy cannot quantitatively determine total uptake, the results show that in the case of the larger AuNRs, more total mass has been taken up by the lung cancer cells compared with that of the smaller AuNRs. This would suggest that the total number of particles may be a more important factor to consider for cellular uptake and targeting than total mass. Consequently, the potential of AuNRs with varied size for cell-related applications such as photoacoustic imaging and photothermal therapy has been demonstrated.

## 4 Conclusions

AuNRs display highly desirable characteristics for use as molecular-targeted contrast agents in photoacoustic imaging and other optical-based diagnostics and therapeutics. It is suggested that there may be an optimal size and concentration that achieves maximum PA emission amplitudes while resisting melting and reshaping, and exhibiting minimal cytotoxicity. We demonstrated that it is important to consider the size of the AuNRs when making a selection for biomedical applications, while also taking into account the concentration of AuNRs at a desired location. At equivalent NP ml^−1^, the smaller AuNRs were shown to exhibit the lowest cytotoxicity to the lung cancer cell line while also displaying the lowest PA emission amplitude, and the larger AuNRs produced the highest PA emission but were the most toxic. Conversely, if the total mass of AuNRs was fixed, then it was the smallest AuNRs that were the most effective PA converters, while the other three sizes produced similar responses. Size-dependent AuNR influences on the melting and reshaping thresholds were also demonstrated, indicating that careful consideration must be made with regards to the laser fluence. The smallest AuNRs (Au10s) displayed the most resistance to melting over the fluence range studied, suggesting the increased photostability of small AuNRs (<25 nm). Dark-field microscopy demonstrated that it may be more important to consider total number of particles when studying cellular uptake and targeting since larger AuNRs resulted in a larger total mass taken up by the cells. The presented study has shown that the size of AuNRs is an important aspect to consider when choosing AuNRs for biomedical uses since it has a strong effect on many crucial characteristics, such as PA emission amplitude, photostability, cellular uptake, and cell toxicity.

## Conflicts of interest

There are no conflicts to declare.

## Supplementary Material
